# *Trichinella* spp. biomass has increased in raccoon dogs (*Nyctereutes procyonoides*) and red foxes (*Vulpes vulpes*) in Estonia

**DOI:** 10.1186/s13071-017-2571-0

**Published:** 2017-12-16

**Authors:** Age Kärssin, Liidia Häkkinen, Enel Niin, Katrin Peik, Annika Vilem, Pikka Jokelainen, Brian Lassen

**Affiliations:** 1 Veterinary and Food Laboratory, Tartu, Estonia; 2 Institute of Veterinary Medicine and Animal Sciences, Estonian University of Life Sciences, Tartu, Estonia; 3 Veterinary and Food Board, Tallinn, Estonia; 4 University of Helsinki, Helsinki, Finland; 5 Statens Serum Institut, Copenhagen, Denmark; 6 Department of Veterinary and Animal Sciences, University of Copenhagen, Frederiksberg C, Denmark

**Keywords:** *Trichinella* infection, *Trichinella nativa*, *Trichinella britovi*, Prevalence, Sylvatic, Zoonosis

## Abstract

**Background:**

Raccoon dogs and red foxes are well-adapted hosts for *Trichinella* spp. The aims of this study were to estimate *Trichinella* infection prevalence and biomass and to investigate which *Trichinella* species circulated in these indicator hosts in Estonia.

**Methods:**

From material collected for evaluating the effectiveness of oral vaccination program for rabies eradication in wildlife, samples from 113 raccoon dogs and 87 red foxes were included in this study. From each animal, 20 g of masseter muscle tissue was tested for the presence of *Trichinella* larvae using an artificial digestion method. The *Trichinella* larvae were identified to species level by multiplex polymerase chain reaction method.

**Results:**

The majority of tested animals were infected with *Trichinella* spp. The parasite species identified were *T. nativa* and *T. britovi.* The apparent infection prevalence was 57.5% in raccoon dogs and 69.0% in red foxes, which were higher than previous estimates. In addition, the larval burden had also increased in both hosts. We estimated that in 2011–2012, the *Trichinella* spp. biomass was more than 15 times higher in raccoon dogs and almost two times higher in red foxes than in 1992–2000 (based on mean larval burden), and almost 20 times higher in raccoon dogs and almost five times higher in red foxes than in 2000–2002 (based on median larval burden).

**Conclusions:**

Raccoon dogs and red foxes are relevant reservoirs for *Trichinella* spp. in Estonia. The biomass of *Trichinella* circulating in sylvatic cycles was substantial and had increased: there is substantial infection pressure in the sylvatic cycle.

## Background

*Trichinella* spp. are zoonotic parasitic nematodes transmitted by carnivorism. Sylvatic *Trichinella* infections are endemic in Estonia, a EU country located in north-eastern Europe that is bordered by Latvia in the south and Russia in the east [1–5]. For example, while anti-*Trichinella* antibodies were not detected in the domestic pigs investigated in our recent study, a substantial proportion of hunted wild boars were *Trichinella* seropositive [5]. Assessment of the sylvatic component and awareness about it are important because there is a risk of spill-over to domestic animals and humans [5].

The raccoon dog (*Nyctereutes procyonoides*) is a suitable indicator species and well-adapted reservoir host for all four *Trichinella* species circulating in Europe, and the red fox (*Vulpes vulpes*) particularly for *T. spiralis* and *T. britovi* [4, 6–10]. The invasive raccoon dog [11], and the native red fox are common and numerous sylvatic carnivores in Estonia [12]. A total of 12,577 raccoon dogs and 7144 red foxes were hunted in Estonia during the hunting season 2011–2012 [13].

The most recent epidemiological data on *Trichinella* infections in raccoon dogs and red foxes in Estonia were based on material collected in 2000–2002 [4]. The apparent prevalence of *Trichinella* spp. was 42.0% in raccoon dogs and 40.6% in red foxes, which did not differ significantly from estimates from 1992 to 2000 [14]. In both earlier studies, *T. nativa* and *T. britovi* were identified in the target hosts. Our study aimed to update the *Trichinella* infection prevalence estimates in raccoon dogs and red foxes in Estonia and to identify the *Trichinella* species causing the infections. We compared the findings with the two previous estimates and with those reported from other European countries and estimated how the biomass of *Trichinella* has changed in Estonia.

## Methods

For the evaluation of the effectiveness of the oral vaccination program for rabies eradication in wildlife [15], head samples from 1214 raccoon dogs and 625 red foxes were collected from whole Estonian territory (average density 4.3 animals per 100 km^2^) from August 2011 to March 2012. The animals sampled were apparently healthy hunted animals, rabies indicator animals killed due to abnormal behavior near human settlements, and animals killed in traffic or found dead. We could investigate muscle samples from 200 of these heads (113 raccoon dogs and 87 red foxes), which was evaluated to be a sufficient sample size to estimate the infection prevalence with 80% confidence level.

To obtain a geographically representative sample for this study, the number of samples from each county was adjusted according to the surface area of the county, and a random sample was drawn from the samples available from there. Data on the estimated age (less than 1 year old = juvenile, at least 1 year old = adult) and gender of each animal had been collected on the submission forms. Age group was unknown for 31 animals and gender for 69 animals.

The samples were kept refrigerated until analysis, but few samples were or could have been frozen (*n* = 3 from raccoon dogs and *n* = 2 from foxes arrived frozen). From each animal, 20 g of masseter muscle tissue was analyzed for the presence of *Trichinella* spp. larvae using the European Union reference method, i.e. magnetic stirrer method, for artificial digestion [16]. The mean time between sampling and digestion was ten days (range: 1–92 days).

Larvae from each positive sample were evaluated morphologically and then counted, rinsed with water, collected, and stored in ethanol at room temperature until identification to species level. The species of *Trichinella* were identified using a previously described multiplex polymerase chain reaction method [17].

The sample size assessment and preliminary statistical calculations were done with OpenEpi software [18]. The confidence intervals (CI) of the prevalence estimates were calculated using Mid-P exact. Comparisons with the prevalence estimates, by host species and *Trichinella* species, from other European countries and previous Estonian studies were done using two by two tables. Two-tailed *P*-values (Mid-P exact) < 0.05 were considered statistically significant.

Logistic regression models were built with STATA 13.0 (Stata Corporation, College Station, Texas, USA) software for three outcomes: testing positive for *Trichinella* spp., testing positive for *T. nativa*, and testing positive for *T. britovi*. The variables we evaluated were ‘host species’ (raccoon dog or red fox), ‘age’ (juvenile or adult), ‘gender’ (female or male), ‘county’ (the 15 counties included as dummy variables i.e. allocated numbers that do not indicate any particular order), and ‘cause of death’ (whether the animal had been hunted, killed due to abnormal behavior, killed in traffic, or found dead). The variables with *P*-value ≤ 0.25 in univariable analysis were included in a multivariable model, followed by a stepwise backward elimination of those with *P* ≥ 0.05 that did not act as confounders.

*Trichinella* spp. biomass was quantitatively estimated for 1000 host animals and for the hunting bag, using estimate of weight of the host, estimate of proportion of muscle of the host weight (based on information available for small mammals of similar size), point estimate of *Trichinella* spp. prevalence, and mean or median larvae per gram of muscle tissue.

## Results

The majority (62.5%, 125/200, 95% CI: 55.6–69.0) of the animals tested were infected with *Trichinella* spp. (Table 1). The apparent *Trichinella* spp. infection prevalence was 57.5% (65/113, 95% CI: 48.3–66.2) in raccoon dogs and 69.0% (60/87, 95% CI: 58.6–77.7) in red foxes. The prevalence was not significantly higher in red foxes than in raccoon dogs.

**Table 1:** *Trichinella* species identified in raccoon dogs (*Nyctereutes procyonoides*) and red foxes (*Vulpes vulpes*) in 2011–2012 in Estonia

*Trichinella* species	Raccoon dog (*n* = 113)					Red fox (*n* = 87)				
	Positive (*n*)	Prevalence (95% CI)^a^ (%)	% of *Trichinella-*positive (95% CI)	Range of lpg	Mean (median) lpg	Positive (*n*)	Prevalence (95% CI)^a^ (%)	% of *Trichinella*-positive (95% CI)	Range of lpg	Mean (median) lpg
*T. nativa* only	23	20.4 (13.7–29.0)	35.4 (24.5–47.5)	0.5–631.6	158.4 (135.0)	19	21.8 (14.1–31.4)	31.7 (20.9–44.2)	0.1–636.8	82.4 (13.8)
*T. britovi* only	15	13.3 (7.9–20.5)	23.1(14.1–34.5)	0.6–486.0	123.5 (58.8)	23	26.4 (18.0–36.4)	38.3 (26.7–51.1)	0.1–409.5	44.7 (10.5)
*T. nativa* and *T. britovi*	13	11.5 (6.6–18.4)	20.0 (11.6–31.8)	26.2–800.0	209.2 (98.0)	8	9.2 (4.4–16.7)	13.3 (6.4–23.8)	2.3–28.6	9.1 (7.1)
*T. nativa*, total^b^	36	31.9 (23.8–40.9)	55.4 (43.2–67.1)	0.5–800.0	176.7 (130.0)	27	31.0 (22.0–41.3)	45.0 (32.8–57.7)	0.1–636.8	60.7 (8.4)
*T. britovi*, total^b^	28	24.8 (17.5–33.4)	43.1 (31.5–55.3)	0.6–800.0	163.3 (83.2)	31	35.6 (26.1–46.1)	51.7 (39.1–64.1)	0.1–409.5	8.2 (35.5)
Species-level result	51	45.1 (36.1–54.4)	78.5 (67.3–87.2)	0.5–800.0	161.1 (101.4)	50	57.5 (46.9–67.5)	83.3 (72.3–91.2)	0.1–636.8	53.3 (9.4)
No species-level result	14	12.4 (7.2–19.5)	21.5 (12.8–32.8)	0.1–576.0	161.1 (43.6)	10	11.5 (6.0–19.5)	16.7 (8.8–27.7)	0.1–142.9	21.4 (2.4)
Total	65	57.5 (48.3–66.4)	100 (95.5–100)	0.1–800.0	161.1 (98.0)	60	69.0 (58.7–78.0)	100 (95.1–100)	0.1–636.8	48.0 (8.2)

*Abbreviation*: lpg, larvae per gram of muscle tissue

^a^95% confidence interval, Mid-P exact

^b^With this particular *Trichinella* species, either as the only species or in mixed infection

The *Trichinella* species present were successfully identified from 80.8% of the animals that had larvae (Table 1). The success rate of *Trichinella* species identification was 82.7% (91/110, 95% CI: 74.8–89.0) from larvae from samples that were digested within the recommended 21 days after sampling [9], and 66.7% (10/15, 95% CI: 40.8–86.6) from samples stored longer. However, the difference was not significant.

The presence of two sylvatic species, *T. nativa* and *T. britovi*, was confirmed (Table 1)*. Trichinella nativa* was detected as the only species present or in mixed infections in 31.9% of raccoon dogs and 31.0% of red foxes, and *T. britovi* was detected as the only species present or in mixed infections in 24.8% of raccoon dogs and 35.6% of red foxes. Of those animals that hosted *Trichinella* spp. larvae that were determined to the species level, *T. nativa* was detected as the only species present or in mixed infection in 70.6% of raccoon dogs and 54.0% of red foxes. Of those animals that hosted *Trichinella* spp. larvae that were determined to the species level, *T. britovi* was detected as the only species or in mixed infection in 54.9% of raccoon dogs and 62.0% of red foxes. The prevalence of mixed infections had increased in red foxes from the estimate of the previous Estonian study (Table 3) [4].

The apparent *Trichinella* spp. infection prevalences estimated from the samples from 2011 to 2012 were higher than those from 2000 to 2002 in both raccoon dogs and red foxes (Tables 2 and 3) [4]. Moreover, the prevalence estimated from samples from 2011 to 2012 was higher than the one from 1992 to 2000 in red foxes; however, the estimate from 2011 to 2012 did not differ significantly from the estimate from 1992 to 2000 in raccoon dogs (Tables 2 and 3) [14].

**Table 2:** Prevalence of *Trichinella* spp. in raccoon dogs (*Nyctereutes procyonoides*) in European countries and comparison with the present study

Country	Sampling period	Samples (*n*)	Prevalence of *Trichinella* spp. (95% CI) (%)	Prevalence of Tn (total) (95% CI) (%)	Prevalence of Tb (total) (95% CI) (%)	Prevalence of Ts (total) (95% CI) (%)	Prevalence of Tp (total) (95% CI) (%)	Prevalence of mixed infections (95% CI) (%)	Reference
Estonia	2011–2012	113	57.5 (48.3–66.4)	31.9 (23.8–40.9)	24.8 (17.5–33.4)	0.0 (0.0–2.6)	0.0 (0.0–2.6)	11.5 (7.9–20.5)	Present study
Estonia	2000–2002	157	42.0* (34.5–49.9)	15.9** (10.8–22.3)	15.9 (10.8–22.3)	0.0 (0.0–5.1)	0.0 (0.0–5.1)	5.1 (2.4–9.4)	[4]
Estonia	1992–2000	33	45.5 (29.2–62.5)	15.2 (5.8–30.4)	27.3 (14.2–44.2)	0.0 (0.0–8.7)	0.0 (0.2–8.7)	3.0 (0.2–14.1)	[3, 14, 57]
Finland	1999–2005	662	28.1*** (24.8–31.6)						[19]
Finland	1996–1998	199	37.7*** (31.2–44.6)						[8]
Germany	2006–2007	146	4.8*** (2.1–9.3)	0.0*** (0.0–2.0)	0.0*** (0.0–2.0)	4.1* (1.7–8.3)	2.7 (0.9–6.5)	1.4*** (0.2–4.5)	[20]
Germany	2000–2014	1527	1.9*** (1.3–2.7)	0.0*** (0.0–0.2)	0.1*** (0.0–0.3)	1.7 (1.1–2.4)	0.1 (0.0–0.3)	0.0 (0.0–0.2)***	[21]
Latvia	2010–2014	394	37.3*** (32.6–42.2)	2.8*** (1.5–4.8)	35.0* (30.4–39.9)	0.5 (0.1–1.7)	0.0 (0.0–0.8)	3.0*** (1.7–5.1)	[22]
Latvia	2000–2002	17	35.3 (15.7–59.5)						[4]
Lithuania	2001–2006	75	29.3*** (19.9–40.4)						[23]
Lithuania	2000–2002	83	32.5*** (23.1–43.1)	2.4*** (0.4–7.7)	25.3 (16.8–35.5)	4.8* (1.6–11.2)	0.0 (0.0–3.5)	4.8 (1.6–11.2)	[4]
Poland	2012	39	5.1*** (0.9–15.9)	0.0*** (0.0–7.4)	0.0*** (0.0–7.4)	5.1 (0.9–15.9)	0.0 (0.0–7.4)	0.0* (0.0–7.4)	[24]

*Abbreviations*: Tn, *Trichinella nativa*; Tb, *Trichinella britovi*; Ts, *Trichinella spiralis*; Tp, *Trichinella pseudospiralis*

*P < 0.05, **P < 0.01, ***P < 0.001

**Table 3:** Prevalence of *Trichinella* spp. in red foxes (*Vulpes vulpes*) in European countries and comparison with the present study

Country	Sampling period	Samples (*n*)	Prevalence of *Trichinella* spp. (95% CI) (%)	Prevalence of Tn (total) (95% CI) (%)	Prevalence of Tb (total) (95% CI) (%)	Prevalence of Ts (total) (95% CI) (%)	Prevalence of Tp (total) (95% CI) (%)	Prevalence of mixed infections (95% CI) (%)	Reference
Estonia	2011–2012	87	69.0 (58.7–78.0)	31.0 (22.0–41.3)	35.6 (26.1–46.1)	0.0 (0.0–3.4)	0.0 (0.0–3.4)	9.2 (4.4–16.7)	Present study
Estonia	2000–2002	446	40.6*** (36.1–45.2)	16.6** (13.4–20.3)	13.5*** (10.5–16.7)	0.0 (0.0–0.7)	0.0 (0.0–0.7)	2.9*(1.6–4.8)	[4]
Estonia	1992–2000	21	42.9* (23.3–64.3)	14.3 (3.8–34.1)	28.6 (12.5–50.2)	0.0 (0.0–13.3)	0.0 (0.0–13.3)	4.8 (0.2–21.3)	[3, 14, 57]
Austria		1546	1.6*** (1.0–2.3)	0.0*** (0.0–0.2)	1.6*** (1.0–2.3)	0.0 (0.0–0.2)	0.0 (0.0–0.2)	0.0*** (0.0–0.2)	[27]
Belgium	1996–2000	818	0.0*** (0.0–0.4)	0.0*** (0.0–0.4)	0.0*** (0.0–0.4)	0.0 (0.0–0.4)	0.0 (0.0–0.4)	0.0*** (0.0–0.4)	[28]
Denmark	1995–1996	3133	0.1*** (0.0–0.3)						[29]
Denmark	1997–1998	3008	0.0*** (0.0–0.1)	0.0*** (0.0–0.1)	0.0*** (0.0–0.1)	0.0 (0.0–0.1)	0.0 (0.0–0.1)	0.0*** (0.0–0.1)	[29]
Finland	1999–2005	1010	18.7*** (16.4–21.2)						[19]
Finland	1996–1998	158	36.7*** (29.5–44.4)						[8]
France	2006–2009	108	2.8*** (0.7–7.4)	0.0*** (0.0–2.7)	2.8*** (0.7–7.4)	0.0 (0.0–2.7)	0.0 (0.0–2.7)	0.0*** (0.0–2.7)	[29]
France	2006–2008	74	0.0*** (0.0–4.0)	0.0*** (0.0–4.0)	0.0*** (0.0–4.0)	0.0 (0.0–4.0)	0.0 (0.0–4.0)	0.0*** (0.0–4.0)	[31]
Germany	2011–2012	3154	0.3*** (0.2–0.6)						[25]
Germany	2006–2007	100	1.0*** (0.1–4.8)	0.0*** (0.0–3.0)	0.0*** (0.0–3.0)	0.0 (0.0–3.0)	0.0 (0.0–3.0)	0.0*** (0.0–3.0)	[20]
Hungary	2008–2013	3304	2.1*** (1.6–2.6)	0.0*** (0.0–0.1)	1.8*** (1.4–2.3)	0.2 (0.1–0.5)	0.0 (0.0–0.1)	0.0*** (0.0–0.1)	[32]
Hungary	2007–2008	2116	1.7*** (1.2–2.3)	0.0*** (0.0–0.1)	1.4*** (1.0–2.0)	0.2 (0.1–0.5)	0.0 (0.0–0.2)	0.0*** (0.0–0.1)	[33]
Italy	2010–2014	153	8.5*** (4.8–13.8)	0.0*** (0.0–1.9)	8.5*** (4.8–13.8)	0.0 (0.0–1.9)	0.0 (0.0–1.9)	0.0*** (0.0–1.9)	[34]
Italy	2004–2014	480	5.0*** (3.3–7.2)						[35]
Italy	2001–2004	229	3.1*** (1.3–6.0)	0.0*** (0.0–1.3)	3.1*** (1.3–6.0)	0.0 (0.0–1.3)	0.0 (0.0–1.3)	0.0*** (0.0–1.3)	[36]
Italy	2001–2004	227	3.5*** (1.7–6.6)	0.0*** (0.0–1.3)	3.5*** (1.7–6.6)	0.0 (0.0–1.3)	0.0 (0.0–1.3)	0.0*** (0.0–1.3)	[37]
Italy	1997–2003	172	1.2*** (0.2–3.8)						[38]
Ireland	2002	454	0.9*** (0.3–2.1)	0.0*** (0.0–0.7)	0.0*** (0.0–0.7)	0.9 (0.3–2.1)	0.0 (0.0–0.7)	0.0*** (0.0–0.7)	[39]
Latvia	2010–2014	668	50.6** (46.8–54.4)	1.5*** (0.8–2.7)	40.9 (37.2–44.6)	0.0 (0.0–0.4)	0.0 (0.0–0.4)	1.0*** (0.5–2.1)	[22]
Latvia	2000–2002	1112	28.9*** (26.3–31.6)	2.2*** (1.4–3.1)	10.3*** (8.6–12.1)	0.4 (0.2–1.0)	0.0 (0.0–0.3)	1.3*** (0.7–2.1)	[4]
Lithuania	2001–2006	206	46.6*** (39.9–53.4)						[23]
Lithuania	2000–2002	567	40.0*** (36.1–44.1)	0.9*** (0.3–1.9)	23.3* (19.9–26.9)	4.8* (3.2–6.8)	0.2 (0.0–0.9)	3.2* (2.0–4.9)	[4]
Netherlands	2010–2013	369	0.3*** (0.0–1.3)						[40]
Netherlands	1996–1997	276	4.0*** (2.1–6.8)	0.0*** (0.0–1.1)	4.0*** (2.1–6.8)	0.0 (0.0–1.1)	0.0 (0.0–1.1)	0.0*** (0.0–1.1)	[41]
Norway	1994–1995, 2002–2005	393	4.8*** (3.0–7.3)	4.6*** (2.8–7.0)	0.3*** (0.0–1.2)	0.0 (0.0–0.8)	0.0 (0.0–0.8)	0.0*** (0.0–0.8)	[42]
Poland	2010–2015	1447	10.0*** (8.6–11.7)	0.0*** (0.0–0.2)	7.2*** (5.9–8.6)	1.1 (0.7–1.8)	0.1 (0.0–0.3)	0.0*** (0.0–0.2)	[26]
Poland	2011–2012	1634	2.7*** (2.0–3.6)	0.1*** (0.0–0.3)	2.0*** (1.4–2.7)	0.6 (0.3–1.0)	0.0 (0.0–0.2)	0.1*** (0.0–0.3)	[25]
Portugal	2008–2010	47	2.1*** (0.1–10.1)	0.0*** (0.0–6.2)	2.1*** (0.1–10.1)	0.0 (0.0–6.2)	0.0 (0.0–6.2)	0.0*** (0.0–6.2)	[43]
Romania	2012–2014	121	21.5*** (14.9–29.5)	0.0*** (0.0–2.4)	19.8* (13.5–27.7)	0.8 (0.0–4.0)	0.0 (0.0–2.4)	0.0*** (0.0–2.4)	[44]
Romania	2000–2005	71	7.0*** (2.6–14.9)	0.0*** (0.0–4.1)	5.6*** (1.8–13.0)	1.4 (0.1–6.7)	0.0 (0.0–4.1)	0.0** (0.0–4.1)	[45]
Serbia	2009–2010	57	12.3*** (5.5–22.8)	0.0*** (0.0–5.1)	3.5*** (0.6–11.1)	8.8** (3.3–18.4)	0.0 (0.0–5.1)	3.5 (0.6–11.1)	[46]
Slovakia	2000–2007	5270	11.5*** (10.7–12.4)						[47]
Slovakia	2007	601	20.3*** (17.2–23.7)	0.0*** (0.0–0.5)	20.3*** (17.2–23.7)	0.3 (0.1–1.1)	0.2 (0.0–0.8)	0.5*** (0.1–1.4)	[47]
Slovakia	2000–2006	4669	10.4*** (9.5–11.3)	0.0*** (0.0–0.1)	8.3*** (7.6–9.2)	0.1 (0.0–0.2)	0.0 (0.0–0.1)	0.0*** (0.0–0.1)	[48]
Slovakia	2000	545	6.1*** (4.3–8.3)	0.0*** (0.0–0.5)	6.1*** (4.3–8.3)	0.0 (0.0–0.5)	0.0 (0.0–0.5)	0.0*** (0.0–0.5)	[47]
Spain	–	400	15.5*** (12.2–19.3)		15.3*** (12.0–19.0)				[49]
Spain	1997–1999	67	8.9*** (3.7–17.7)						[50]
Spain	1985–1997	227	2.6*** (1.1–5.4)	0.0*** (0.0–1.3)	1.8*** (0.6–4.2)	0.9 (0.1–2.9)	0.0 (0.0–1.3)	0.0*** (0.0–1.3)	[51]
Spain	1989–1993	84	1.2*** (0.1–5.7)						[52]
Sweden	1985–2003	1800	4.5*** (3.6–5.5)						[53]
Switzerland	2006–2007	1289	1.6*** (1.0–2.4)	0.0*** (0.0–0.2)	1.6*** (1.0–2.4)	0.0 (0.0–0.2)	0.0 (0.0–0.2)	0.0*** (0.0–0.2)	[54]
United Kingdom (Great Britain)	2003–2007	1144	0.0*** (0.0–0.3)	0.0*** (0.0–0.3)	0.0*** (0.0–0.3)	0.0 (0.0–0.3)	0.0 (0.0–0.3)	0.0*** (0.0–0.3)	[55]
United Kingdom (Northern Ireland)	2003–2004; 2006–2007	443	0.2*** (0.0–1.1)	0.0*** (0.0–0.7)	0.0*** (0.0–0.7)	0.2 (0.0–1.1)	0.0 (0.0–0.7)	0.0*** (0.0–0.7)	[56]

*Abbreviations*: Tn, *Trichinella nativa*; Tb, *Trichinella britovi*; Ts, *Trichinella spiralis*; Tp, *Trichinella pseudospiralis*

*P < 0.05, **P < 0.01, ***P < 0.001

The number of *Trichinella* spp. larvae recovered per gram muscle tissue (lpg) was higher in raccoon dogs (median: 98.0, mean: 161.1, range: 0.1–800.0 lpg) than in red foxes (median: 8.2, mean: 48.0, range: 0.1–636.8 lpg), and varied by *Trichinella* species (Table 1). The highest larval burden, 800 lpg, was detected in a raccoon dog with mixed infection.

The median larval burden had increased in both raccoon dogs and red foxes from those reported in the previous study: from 7.2 lpg to 98.0 lpg in raccoon dogs and from 3.0 lpg to 8.2 lpg in red foxes [4]. Furthermore, the proportion of animals with low larval burden (< 1 lpg) had decreased from 18.1% to 7.7% in raccoon dogs, and from 23.7% to 11.7% in red foxes, further indicating that the circulating parasite biomass of *Trichinella* larvae had increased [4]. The *Trichinella* spp. biomass was estimated to have increased 18.6-fold in raccoon dogs and 4.6-fold in red foxes (based on median larval burden) (Table 4).

**Table 4:** Calculation of the change in *Trichinella* spp. biomass in raccoon dogs and red foxes in Estonia

	Raccoon dog	Red fox	Reference
Hunting bag 1995, *n* animals	1723	3326	[67]
Hunting bag 2001, *n* animals	4259	6628	[67]
Hunting bag 2011, *n* animals	12,577	7144	[13]
With *Trichinella* larvae (%)			
1992–2000	45.5	42.9	[14]
2000–2002	42.0	40.6	[4]
2011–2012	57.5	69.0	Present study
Mean body weight of host, g	4830	4890	[68, 69]
Muscle tissue of body weight, %	60	60	[70]
Median (mean) *Trichinella* lpg			
1992–1996	nd (13.4)	nd (43.1)	[2]
2000–2002	7.2 (nd)	3.0 (nd)	[4]
2011–2012	98.0 (161.1)	8.2 (48.0)	Current study
*Trichinella* biomass 1992–2000, median (mean) *n* larvae			
in 1000 animals	nd (17,669,106)	nd (54,249,367)	
in the hunting bag^a^	nd (30,443,870)	nd (180,433,393)	
*Trichinella* biomass 2000–2002, median (mean) *n* larvae			
in 1000 animals	8,763,552 (nd)	3,573,612 (nd)	
in the hunting bag^b^	37,323,968 (nd)	23,685,900 (nd)	
*Trichinella* biomass 2011–2012, median (mean) *n* larvae			
in 1000 animals	163,302,300 (268,448,985)	16,600,572 (97,174,080)	
in the hunting bag^c^	2,053,853,027 (3,376,282,884)	118,594,486 (694,211,628)	
Increase in *Trichinella* biomass from 1992 to 2000 to 2011–2012, calculated from median (mean) *n* larvae			
in 1000 animals	nd (15.2-fold)	nd (1.8-fold)	
in the hunting bag	nd (110.9-fold)	nd (3.8-fold)	
Increase in *Trichinella* biomass from 2000 to 2002 to 2011–2012, calculated from median (mean) *n* larvae			
in 1000 animals	18.6-fold (nd)	4.6-fold (nd)	
in the hunting bag	55.0-fold (nd)	5.0-fold (nd)	

*Abbreviations*: nd, no data; lpg, larvae per gram of muscle tissue

^a^ *n* larvae = *n* animals 1995 × % with larvae 1992–2000 × (mean body weight of host, g × muscle tissue of body weight, %) × median (mean) lpg (1992–1996)

^b^ *n* larvae = *n* animals 2001 × % with larvae 2000–2002 × (mean body weight of host, g × muscle tissue of body weight, %) × median (mean) lpg (2000–2002)

^c^ *n* larvae = *n* animals 2011 × % with larvae 2011–2012 × (mean body weight of host, g × muscle tissue of body weight, %) × median (mean) lpg (2011–2012)

**Fig. 1:**
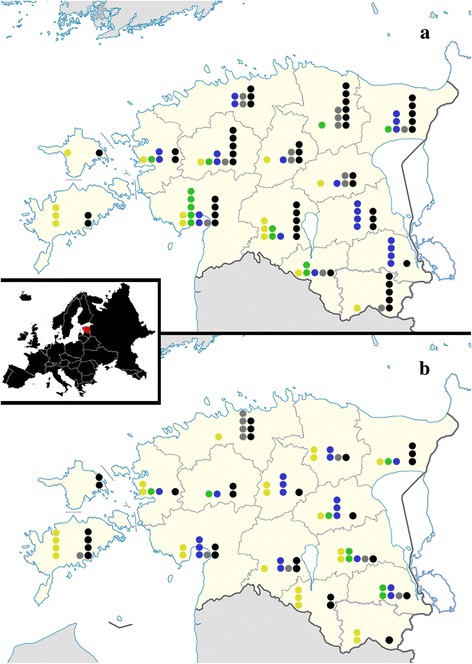
*Trichinella* spp. in raccoon dogs (*Nyctereutes procyonoides*) (**a**) and red foxes (*Vulpes vulpes*) (**b**) in 2011–2012 in Estonia, by counties. *Key*: yellow dot, *T. britovi*; green dot, *T. britovi + T. nativa*; blue dot, *T. nativa*; grey dot, *Trichinella* spp. (no species-level result); black dot, no larvae detected

*Trichinella nativa* was not detected in samples from the large islands Saaremaa and Hiiumaa, nor the most southeastern county Võrumaa, while *T. britovi* was found in samples collected from all counties (Fig. 1).

None of the variables were significant factors for testing positive for *Trichinella* spp. in either of the hosts nor in both hosts together. The final model for testing positive for *T. nativa* had two variables, ‘age’ and ‘county’, and the area under the receiver operating characteristic (ROC) curve was 0.72. The odds of testing positive for *T*. *nativa* were 3.6 times (*P* = 0.009, 95% CI: 1.4–9.3) higher in adults than in juveniles, and higher in counties Põlvamaa and Pärnumaa when compared with Harjumaa where the capital is located (*P* = 0.009, OR = 15.2, 95% CI: 2.0–117.3, and *P* = 0.029, OR = 7.6, 95% CI: 1.2–47.1, respectively). The final model for testing *T. britovi* positive included only the variable ‘county’, and the area under the ROC curve was 0.68. The odds of an animal testing *T. britovi* positive were higher in the counties Valgamaa, Saaremaa, Läänemaa, and Pärnumaa (*P* = 0.019, OR = 16.8, 95% CI: 1.6–176.2; *P* = 0.023, OR = 14.0, 95% CI: 1.4–137.3; *P* = 0.040, OR =11.7, 95% CI: 1.1–122.4; and *P* = 0.043, OR = 9.7, 95% CI: 1.1–87.4, respectively) than in the reference county Harjumaa.

## Discussion

We summarized the results of European studies on *Trichinella* spp. infection prevalence in raccoon dogs (Table 2) and red foxes (Table 3). Lower prevalences than our estimate from Estonia have been observed in both hosts in Finland, Latvia, Lithuania, Poland and Germany (Tables 2 and 3) [19–26]. Moreover, in red foxes, the *Trichinella* spp. infection prevalence was higher in Estonia than what has been reported in Austria, Belgium, Denmark, France, Great Britain, Hungary, Italy, Ireland, Netherlands, Norway, Northern Ireland, Portugal, Romania, Serbia, Slovakia, Spain and Switzerland (Table 3) [26–56]. However, as different sampling schemes, sample sizes, sample material, and detection methods were used, these studies are not all directly comparable with our study.

In Europe, according to the International *Trichinella* Reference Centre [57], the northern species *T. nativa* has been found in raccoon dogs in Estonia, Finland, Latvia, Russia, and Sweden; and in red foxes in Estonia, Finland, Germany, Latvia, Norway, Poland, Sweden and Ukraine. The published studies on *T. nativa* in raccoon dogs and red foxes report lower prevalences (single and mixed infections included) in Latvia, Lithuania and Norway than our estimate from Estonia [4, 22, 42]. In Poland and Germany, *T. nativa* has been found in red foxes (Table 3) [20, 21, 24–26]. When comparing the result of our study with that from the previous Estonian study, the *T. nativa* infection prevalence had increased in both raccoon dogs and red foxes (Tables 2 and 3) [4].

In Europe, *T. britovi* has been found in raccoon dogs in Estonia, Finland, Germany, Latvia and Lithuania (Table 2) [4, 57]. It is the most common *Trichinella* species in red foxes in Europe [10]. The prevalence of *T*. *britovi* we observed in raccoon dogs in single and mixed infections was similar to that reported from Lithuania, lower than that from Latvia, and higher than those from western Poland and Germany (Table 2) [4, 21, 22, 24]. The prevalence of *T. britovi* we observed in red foxes, including both single and mixed infections, was higher than those reported from Austria, France, Hungary, Norway, Poland, Portugal, Romania, Serbia, Slovakia and Switzerland (Table 3) [21–24, 32, 33, 45–48, 50, 54–56]. A similar to our prevalence estimate for *T. britovi* was detected in red foxes in Latvia [22]. When comparing the result of our study with that from the previous Estonian study, the *T. britovi* infection prevalence had increased in red foxes (Table 3) [4]. Moreover, mixed infections were more common in our study than what was observed in raccoon dogs and red foxes in the neighboring country Latvia and in red foxes in Lithuania (Tables 2 and 3) [4, 22].

In this study, the odds of being *Trichinella*-infected were not significantly different in raccoon dogs and red foxes, whereas the mean larval burden was 3.2 times higher in raccoon dogs than in red foxes. In Latvia, red foxes had higher odds to test positive *(P* = 0.010, OR = 1.41, 95% CI: 1.08–1.83) than raccoon dogs, but raccoon dogs had 2.9 times higher mean larval burden than red foxes [22]. In Finland, both indicators were higher in raccoon dogs than in red foxes (*P* < 0.001, OR = 1.70, 95% CI: 1.35–2.14; 3.8 times higher mean larval burden) [19]. A higher larval burden in raccoon dogs than in red foxes has also been described in other studies [4, 8].

Despite the fact that we used 20 g of tissue for the digestion, our study likely underestimated the actual infection prevalence and larval burdens, because the available material was not optimal for finding *Trichinella* larvae [9, 58, 59]. In experimentally infected raccoon dogs, the *T. nativa* larval density in masseter muscle was about half of that in foreleg muscles [58]. The storage conditions and transport time could also affect the results [29, 40, 60].

The high *Trichinella* infection prevalence in raccoon dogs and red foxes, as well as the overall circulation of the parasites in the sylvatic cycle, may be supported by human behavior. For example, the local hunters use carcasses of hunted raccoon dogs as baits [61], which might help the transmission. According to winter tracking index and hunters’ estimations, after the rabies vaccination program started in 2005 [62], the red fox population size first increased, with a peak in 2009–2010, and then decreased [12]. The raccoon dog population size has increased since the second half of last century [62] and has relatively stabilized after 2011–2012 [12]. These changes are also reflected in the increased hunting bag sizes [13] and may have relevance beyond simply higher numbers. There was an association between the abundance index of raccoon dogs and the proportion of *Trichinella*-infected raccoon dogs and red foxes in Finland [19].

Estonia is located in the transition zone of maritime and continental climate [63]. The coldest months with mean air temperature below zero are December to February [64]. According to data covering these three months from six weather stations located in Harjumaa, Lääne-Virumaa, Pärnumaa, Saaremaa, Tartumaa and Võrumaa, the mean number of days with snow cover was 12.7% (from 4% in Pärnumaa to 26% in Saaremaa) higher in 2002–2011 than in 1992–2001 (data received on request from Estonian Environment Agency). The snow cover could reduce the destructive effect of freezing-thawing cycles on carcasses of infected animals and thus facilitate survival of *Trichinella* larvae [65, 66].

Raccoon dogs and red foxes act as reservoir hosts for *Trichinella* spp. in the sylvatic cycle, where the infection can spread to game animals, such as wild boars, that are hunted for human consumption. The *Trichinella* seroprevalence in wild boars is high in Estonia [5], and the odds of testing *Trichinella*-seropositive were higher if the wild boar was hunted in certain counties, including Pärnumaa and Saaremaa, when compared with Harjumaa. In this study, a similar comparison was made, with Harjumaa as the reference county. Raccoon dogs and red foxes had higher odds to test *T. nativa* positive in Pärnumaa, whereas the odds to test *T. britovi* positive were higher in Pärnumaa and Saaremaa. Moreover, the highest larval burden was detected in a young raccoon dog from Pärnumaa. This raccoon dog had a mixed infection. These two counties could thus be interesting for further studies.

We estimated that in 2011–2012, the *Trichinella* spp. biomass was more than 15 times higher in raccoon dogs and almost two times higher in red foxes than in 1992–2000 (based on mean larval burden), and almost 20 times higher in raccoon dogs and almost five times higher in red foxes than in 2000–2002 (based on median larval burden) (Table 4). Using the increased hunting bag in the calculation as an indication of increased population size or as an indication of biomass removed from the circulation by hunting, the role of these hosts as reservoirs was clearly illustrated (Table 4). The widespread distribution of *Trichinella* infections in Estonian wildlife underlines that there is a high infection pressure within the eastern European sylvatic cycles. Moreover, the results of this study indicate that there is an increase in the infection pressure. *Trichinella* spp. thrive in Estonia, and there is a continuous risk of spill-over to domestic animals and humans.

## Conclusions

In Estonia, the proportion of both raccoon dogs and red foxes that hosted *Trichinella* were higher than ten years earlier. In addition, the larval burdens had also increased in these hosts, and an increased biomass of *Trichinella* larvae was circulating in sylvatic cycles. *Trichinella nativa* and *T. britovi* were found in both host species. There is a substantial and increasing *Trichinella* infection pressure to the food chains and humans.

## Abbreviations

CI: confidence interval
nd: no data
OR: odds ratio
ROC curve: receiver operating characteristic (ROC) curve
Tb: *Trichinella britovi*
Tn: *Trichinella nativa*
Tp: *Trichinella pseudospiralis*
Ts: *Trichinella spiralis*
